# Hollow-core polydopamine nanocarriers for ultrasound-enhanced drug delivery

**DOI:** 10.1039/d5nh00297d

**Published:** 2025-11-06

**Authors:** Swetha Lingamgunta, Chitra Yadav, Andrea Orthodoxou, Lauren Gilmour, Matthew Ellis, Hildegard Metzger, Andrea Bistrovic Popov, Helen Mulvana, Ljiljana Fruk

**Affiliations:** a Department of Chemical Engineering and Biotechnology, University of Cambridge, Philippa Fawcett Drive Cambridge CB3 0EL UK lf389@cam.ac.uk; b James Watt School of Engineering, University of Glasgow Glasgow UK

## Abstract

On-demand drug release is one of the main challenges in nanocarrier design and a key step toward enhancing the efficacy of novel therapeutic formulations. Compared to conventional methods such as pH- or light-driven release, ultrasound-guided drug release offers a cost-effective strategy with improved tissue penetration making it particularly suitable for applications in hard-to-access tissues such as the pancreas. In this study, hollow nanoparticles (hPDA) were developed and evaluated for ultrasound-enhanced drug delivery, focusing on pancreatic ductal adenocarcinoma (PDAC). The hPDA nanoparticles, prepared employing non-toxic reagents, measured approximately 120 nm and were successfully loaded with SN-38, a potent yet challenging-to-formulate chemotherapeutic agent. Ultrasound-triggered drug release experiments at 60 kHz and 1.1 MHz demonstrated significant enhancements in drug release, with an increase of 54% and 19% respectively, compared to controls. Cytotoxicity studies under ultrasound exposure revealed a 20% reduction in cell viability, underscoring the synergistic potential of hPDA and ultrasound technology. These findings establish hPDA nanocarriers as a promising platform for ultrasound-responsive, targeted drug delivery in cancer therapy, with high potential for improved spatiotemporal control and reduced systemic toxicity.

## Abstract 2

Hollow core polydopamine nanoparticles are used for encapsulation and ultrasound-aided release of a potent chemotherapeutic drug.

## Graphical abstract

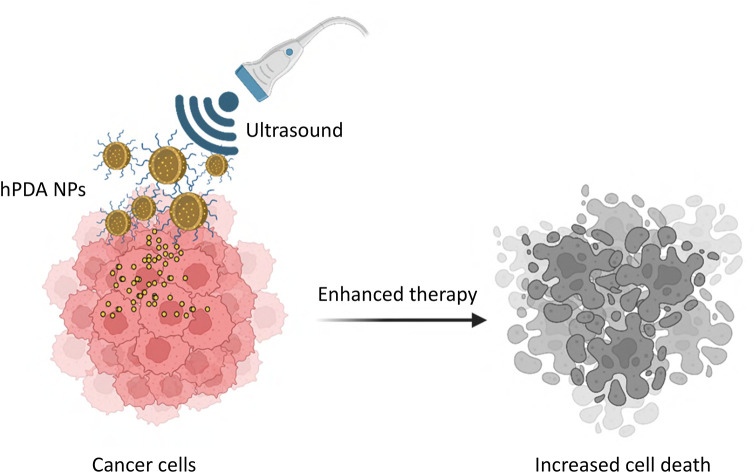

**New concepts**

Pancreatic ductal adenocarcinoma (PDAC) remains among the most difficult-to-treat cancers because of its fibrotic stroma and poor vascularisation that hinder drug penetration, and resistance to standard therapies. In response to the urgent need for more efficient therapies, we have developed hollow polydopamine nanoparticles (hPDA NPs) that release their payload in response to clinically relevant ultrasound (1.1 MHz). Unlike pH-sensitive or photothermally-aided drug delivery, ultrasound offers a mild and inexpensive strategy that is already integrated into routine clinical imaging. Through a fully biocompatible, scalable synthesis, we produced the first hollow core PDA NPs that combine the high drug-loading capacity and sub-200 nm size of polymeric carriers with the dynamic acoustic responsiveness normally reserved for microbubbles. High-speed optical imaging confirmed robust cavitation of the hollow cores under ultrasound, resulting in an enhanced drug release and cytotoxic efficacy compared with solid PDA controls. This platform offers a non-invasive, spatiotemporally controllable strategy to enhance chemotherapeutic delivery in PDAC and other stroma-rich solid tumours.

## 1. Introduction

Solid tumours such as pancreatic ductal adenocarcinoma (PDAC) remain among the most challenging cancers to treat, largely due to their complex tumour microenvironment, which supports cancer growth, reduces drug efficacy, and shields malignant cells from immune surveillance.^1,2^ This immunosuppressive nature is further intensified by genetic mutations, metabolic dysregulation, and chronic inflammation, factors that collectively drive resistance to both conventional treatments and immuotherapies.^3^ As a result, the 5-year survival rate for PDAC remains below 9%.^3^

Alarmingly, recent data indicate a rising incidence of PDAC in younger populations, particularly among women. In the UK, between 1993 and 2018, cases increased by 208% in females aged 0–24 years and by 34% in those aged 25–49 years.^4^ Unlike many other cancers, the treatment of PDAC over the past two decades has been limited progress, with surgical resection still the most effective intervention. For inoperable cases, standard care involves chemotherapy, either gemcitabine monotherapy or the more potent but highly toxic four-drug regiment FOLFIRINOX.^5,6^ However, both approaches are hampered by poor drug delivery to the tumour site and lack of specificity.

To improve the solubility of hydrophobic drugs and enhance tumour penetration and delivery,^7^ a range of nanocarrier systems have been developed. Notably, albumin-bound paclitaxel (Abraxane)^8^ and liposomal irinotecan (Onivyde)^9^ have received FDA approval. While these first-generation nanocarriers provided more stable drug formulations, they failed to yield substantial improvements in overall survival. This underscores the need for nanocarriers that not only carry potent agents but also enable precise, on-demand drug release within solid tumours.

Stimuli-responsive nanocarriers triggered by pH,^10,11^ enzymatic activity, intrinsic redox agents,^12^ light,^13^ magnetic field^14^ or ultrasound^15^ have been extensively explored in past decades. Among these, ultrasound stands out as a non-invasive, versatile, and cost-effective approach with superior penetration depth compared to other external activation methods.^16^

Ultrasound can be broadly classified into low-intensity ultrasound (LIUS) (<3 W cm^−2^),^17^ and high-intensity focused ultrasound (HIFU) (≥5 W cm^−2^).^18,19^ LIUS is widely regarded as extremely safe, with minimal risk of soft tissue damage even during prolonged exposure.^20^ These safety profiles are supported by decades of research, including extensive *in vitro* assessment of cell viability.^21^ Despite its low power, LIUS can enhance *in vivo* circulation, and in conjunction with ultrasound contrast agent microbubbles, has been shown to temporarily increase cell membrane and tissue permeability, properties that make it highly attractive for responsive drug delivery applications.^22,23^ LIUS is generally characterised by longer wavelengths, lower spatial resolution, and larger treatment zones that HIFU, and is also used in therapy, for example to stimulate bone healing in non/union fractures. In contrast, HIFU enables localised therapy with minimal side effects by precisely focusing ultrasound on the target site, facilitating controlled, site-specific drug release.^21,24^

Microbubbles (MBs), widely used as contrast agents in diagnostic ultrasound, have also shown considerable potential in therapeutic applications, including drug and gene delivery, tumour ablation, and blood–brain barrier (BBB) opening.^16,25^ By amplifying localised forces, MBs reduce the ultrasound pressure required to induce mechanical effects on cells and tissues. Upon ultrasound application, MBs oscillate, generating phenomena such as microstreaming, in which shear forces capable of acting over distances of several orders of magnitude greater than the MB diameter (*e.g.*, >100 μm), can transiently disrupt the plasma membrane and stimulate endocytosis.^26^ At higher ultrasound intensities, MB collapse or cavitate, producing shock waves. When cavitation occurs near a cell surface, the resulting micro-jets can puncture the membrane, thereby facilitating intracellular delivery of therapeutic agents. Typically, such applications employ low ultrasound frequencies within the range used in medical imaging (1–3 MHz). Much lower frequencies (20–60 kHz) have also been investigated, particularly for intestinal delivery or for the administration of large molecular weight pharmaceuticals.^27^

A major limitation of MB-based systems is their relatively large size (1–10 μm) and the presence of the unstable gas core, which limits their use to intravascular applications, as they are unable to extravasate into surrounding tissues.^28^ Their short *in vivo* circulation time, typically on order of minutes, further limits efficacy, as rapid gas core dissipation reduces their potential for sustained drug delivery.^21^

**Fig. 1:**
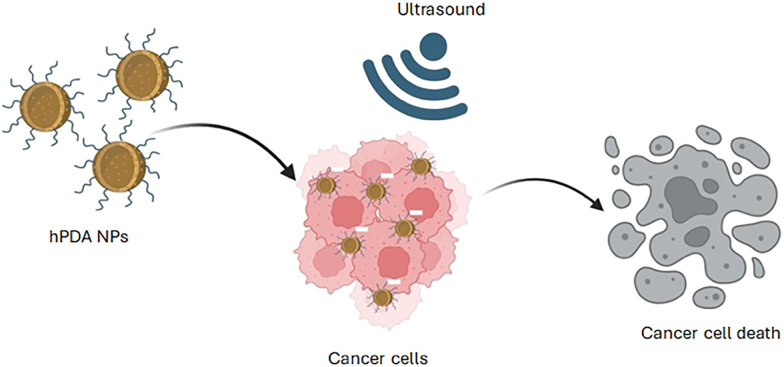
Hollow polydopamine nanoparticles (hPDA NPs) loaded with chemotherapeutic can be used for ultrasound-induced drug release and cell death.

To address these limitations of conventional ultrasound responsive systems, and improve cytotoxic efficacy through on-demand drug release through acoustic cavitation, we developed an ultrasound-responsive nanosized hollow polydopamine (hPDA) nanocarrier (Fig. 1). Synthesised from the melanin-like polymer polydopamine, PDA nanoparticles (NPs) are inherently biocompatible and have been shown to scavenge free radicals and confer neuroprotection.^29–31^ In addition, PDA NPs possess exceptional photothermal and photoacoustic properties, making them highly attractive for applications in drug delivery,^32,33^ imaging,^34,35^ and diagnostics.^36^

Recently, we reported the preparation of polydopamine (PDA)-pluronic structures with tuneable sizes, which were successfully loaded with a potent chemotherapeutic drug, 7-ethyl-10-hydroxycamptothecin (SN-38). These nanostructures exhibited low immunogenicity and high uptake across multiple PDAC cell lines.^37^ The abundant amino and catechol groups present in PDA enable facile conjugation of functional molecules to its surface.^38^ Furthermore, the aromatic backbone of PDA facilitates the encapsulation of hydrophobic drugs through π–π stacking interactions and hydrophobic nature of PDA matrix.^39^ The drug-loading capacity of PDA NPs can be further enhanced by increasing their porosity, thereby significantly increasing the available surface area for drug adsorption. Studies have demonstrated that mesoporous PDA exhibits markedly higher drug-loading efficiency compared to non-porous PDA, owing to the synergistic effects of π–π and hydrophobic interactions, as well as multilayer adsorption mechanisms.^40,41^

Porous PDA NPs are commonly synthesised using templating strategies during the oxidative polymerisation of dopamine.^38,42,43^ In a typical approach, emulsion droplets of 1,3,5-trimethylbenzene (TMB) stabilized with triblock copolymers, or high-molecular-weight block copolymer micelles, serve as sacrificial templates.^44,45^ Dopamine polymerises around these templates, forming a porous PDA shell. Subsequent removal of the templating agents yields mesoporous PDA structures with increased surface area and improved drug-loading capacity.

Traditional porous PDA systems rely on toxic TMB precursors, which limit large-scale production and raise biocompatibility concerns. Our hollow, ultrasound-responsive PDA nanoparticles overcome these limitations by employing entirely biocompatible precursors (umbelliferone and dopamine), while leveraging their hollow structure and acoustic cavitation to achieve precise, on-demand drug release. Compared with solid PDA nanoparticles, this platform demonstrates enhanced therapeutic efficacy, including higher drug loading, tunable release kinetics, and increased cytotoxicity against cancer cells. By combining nanoscale dimensions, biocompatibility, and ultrasound responsiveness, these nanoparticles offer a clinically relevant strategy for efficient, targeted, and spatiotemporally controlled cancer therapy.

## 2. Experimental section

### 2.1. Materials and characterisation methods

All materials were purchased from either Acros Organics, Alfa Aeser or Sigma-Aldrich in the highest purity available and used without further purification. Cell lines (BxPC-3 and PANC-1) were purchased from the American Type Culture Collection (ATCC). ^1^H and ^13^C NMR measurements were carried out using a 500 MHz DCH cryoprobe spectrometer. UV-vis absorption spectra were obtained with an Agilent Cary 300 spectrophotometer. DLS and Zeta Potential measurements were recorded using a Zetasizer nano range instrument (Malvern Panalytical). SEM images were obtained on a TESCAN MIRA3 FEG-SEM and TEM images on a Thermo Scientific (FEI) Talos F200X G2 TEM. Samples were dispersed in water and drop cast on lacey carbon copper grids (Agar Scientific). Lyophilisation was carried out using a Telstar LyoQuest benchtop freeze dryer (0.008 mBar, −70 °C).

### 2.2. Synthesis of hollow polydopamine nanoparticles (hPDA)

A mixture of umbelliferone (360 mg) and Pluronic F127 (360 mg) in 55 mL of ethanol and 60 mL Milli-Q water was stirred at room temperature for 30 minutes. Subsequently, Tris base (90 mg) dissolved in Milli-Q water (2 mL) was added to the reaction mixture. Dopamine hydrochloride (60 mg in 2 mL of Milli-Q water) was then added dropwise under continuous stirring. The reaction was allowed to proceed for 36 h. After synthesis, the mixture was centrifuged at 14 000 rpm on a Lynx 4000 centrifuge, and the supernatant was discarded. For removal of the micelle template, the resulting pellet was resuspended in an equal mixture of ethanol and acetone, followed by sonication for 30 min. The particles were then washed three times with Milli-Q water *via* centrifugation at 14 000 rpm. The final precipitate was resuspended in Milli-Q water to obtain a final concentration of 2 mg mL^−1^. The concentration of NPs was determined by freeze drying with mannitol as a cryoprotectant.

To improve the stability of the particles for subsequent *in vitro* studies, hPDA NPs were coated with a polyethylene glycol (PEG). Methoxy-PEG amine (PEG 2000) with a ratio of 2 : 1 mPEG-amine: NPs was introduced into the hPDA NP suspension in water (pH ∼8.5) and stirred overnight. The PEG-coated NPs were then washed three times with water to remove unreacted components, yielding stable, PEG-functionalised hPDA NPs for further applications. PEGylation was carried out as the last step before further use, *i.e.*, after drug loading.

The colloidal stability of NPs was assessed in deionised water, PBS (1×, pH = 5.5–8.5), phenol red-free DMEM, and DMEM supplemented with 10% FBS. The pH of PBS was adjusted to using HCl or NaOH. For each condition, 900 μL of solvent was mixed with 100 μL of a 1.0 mg mL^−1^ NP suspension and incubated at 37 °C for 72 h. Following incubation, hydrodynamic diameter, zeta potential, and UV-vis absorbance were measured to evaluate nanoparticle stability.

### 2.3. Kinetics of hPDA nanoparticle formation

During hPDA synthesis, aliquots were collected at regular intervals over 72 h from the reaction mixture and measured by DLS to assess hydrodynamic size. The absorbance measurements were performed using a separate reaction mixture in a cuvette. A custom UV-vis measurement setup was assembled to monitor the synthesis of polydopamine (hPDA) nanocarriers over 72 h as shown in SI, Fig. S1. The setup includes DH-2000 Deuterium–Tungsten Halogen UV-vis-NIR Light Source (Ocean Insight) coupled to an Ocean Optics QE65000 spectrometer, enabling precise spectral analysis throughout the synthesis process.

### 2.4. Photothermal properties of hPDA NPs

The photothermal properties of hPDA and the PDA control were evaluated using a custom-built cuvette-based setup (SI, Fig. 1) and TC-08 thermocouple data logger to record temperature changes. For each sample (2 mg mL^−1^), the temperature was first measured under ambient conditions for 1 min. The cuvette was then irradiated with an 808 nm laser for 5 min, after which the laser was turned off, and temperature measurements continued for an additional 5 min to monitor cooling dynamics

The experiment was performed in triplicate to ensure reproducibility.

### 2.5. Confocal imaging of hPDA uptake

Pancreatic cancer cells (BxPC-3 and PANC-1) were seeded into glass-bottom dishes (MatTek Life Science, US) at a concentration of 200 000 cells per mL and incubated at 37 °C for 24 h. They were then treated with rhodamine-labelled hPDA NPs (Rh@hPDA) at different concentrations for 24 h at 37 °C. Following treatment, cells were washed three times with 1× PBS, then stained with CellMask™ Deep Red Plasma Membrane Stain and Hoechst 33342 (Thermo Fisher) according to the manufacturer's instructions. After staining, cells were gently washed three more times with 1× PBS and imaged using a confocal microscope (Axio Observer Z1 LSM 800, Zeiss). Image acquisition and processing were performed using Zen software (Zeiss). MBs used in the studies were USphere phospholipid shells filled with perfluoropropane (C_3_F_8_) MBs with a size range of 1.1–1.4 μm.

### 2.6. Loading of hPDA with 7-ethyl-10-hydroxycamptothecin (SN38)

hPDA were mixed with SN-38 dissolved in DMSO (4 : 1 wt% ratio) and the reaction was left to proceed overnight. To remove unbound or weakly associated SN-38 molecules, the reaction mixture was subjected to centrifugation at 2000 rpm for 5 min. The supernatant, containing the SN-38@hPDA NPs, was collected, while the resulting pellet, primarily consisting of unbound SN-38 aggregates and excess reactants, was discarded. To ensure thorough removal of free SN-38, the supernatant was subjected to additional washing steps using 2% DMSO followed by repeated centrifugation cycles under the same conditions. The purified SN-38@hPDA NPs were then resuspended in Milli Q water for further characterisation and evaluation. Successful loading and quantification of SN-38 was determined by UV-vis spectroscopy. Loading capacity and efficiency were assessed using following equations and are additionally shown in SI, Table S2:
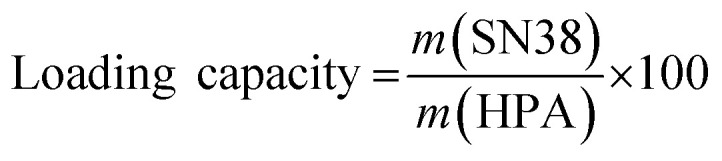

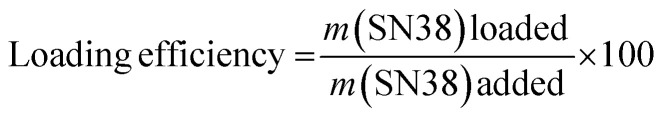


### 2.7. MTS cytotoxicity studies

The effect of SN-38@hPDA NPs on the viability of BxPC-3 and PANC-1 was studied using MTS assay (3-(4,5-dimethylthiazol-2-yl)-5-(3-carboxymethoxyphenyl)-2-(4-sulfophenyl)-2*H*-tetrazolium) (Promega, USA). In addition, healthy pancreatic cell line (pancreatic stallate cell, hPSC) was used to assess the toxicity. Cells were seeded into clear 96-well plates containing 2000 cells per well in 100 μL complete growth medium and cultured for 24 h at 37 °C and 5% CO_2_. Subsequently, cells were treated with varying concentrations of hPDA, SN-38@hPDA, PDA and SN-38@PDA (0.01–100 μg mL^−1^), dissolved in complete growth media containing 0.1% water. After further 72 h incubation at 37 °C and 5% CO_2_, 20 μL of CellTiter 96® Aqueous One Solution (Promega, USA) was added into each well and incubated at 37 °C, 5% CO_2_ for 1–4 h, according to the manufacturer's instruction. The absorbance of each well was measured at 490 nm using a Spark plate reader (TECAN, CH). Control measurements included negative control of cells with DMEM, cells with DMEM containing 0.1% water, cell-free culture media (blank) and cell-free sample dilutions in culture media to evaluate potential sample interferences with MTS reagent. All experiments were conducted in biological triplicates. The percentage cell viability was calculated according to the following:
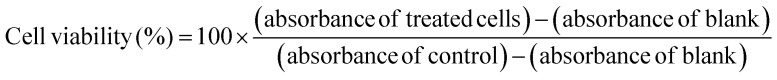


### 2.8. Ultrasound-induced cavitation of hPDA NPs

The study was conducted inside a bespoke 420 × 438 × 220 mm^3^ cavitation tank, filled with deionized and degassed water. A 90 mm diameter transducer (H-198, Sonic Concepts) was excited by a power amplifier (1040L, E&I) and driven for 100 cycles at 1.1 MHz by an arbitrary waveform generator (DG4102, Rigol). A 500 μm polycarbonate capillary (Paradigm Optics) was situated in the centre of the ultrasound focal region *via* a custom 3D printed mount, with its inlet and outlet secured to silicon tubing using epoxy. PDA and hPDA NPs were prepared at a concentration of 1 mg mL^−1^ and flowed through the capillary from a syringe equipped with a 20G microlance, which was inserted into the bore of the silicon tubing inlet. The silicon tubing outlet vented to a collection reservoir outside of the tank.

Cavitation dynamics at the intersection of the ultrasound focus and the capillary were captured using a high-speed camera (HPV-X, Shimadzu) with a 5× objective lens at a frame rate of 10 × 10^6^ frames per second (fps) over a duration of 25.5 μs. The imaging sequence was triggered using a pulse delay generator (DG535, Stanford Research) 51.6 μs after transducer excitation, ensuring sufficient time for the ultrasound wave to propagate from the transducer to the capillary. The range of pressures investigated were 60–100 mVpp (0.9–1.6 MPa/4.6–7.6 mV PkNeg). The peak negative pressure at the focus had been earlier determined through calibrated needle hydrophone measurements (0.2 mm; Precision Acoustics Ltd).

Illumination was provided from below using a liquid light guide synchronised with 10 ns laser pulses (CAVILUX, Cavitar). To confirm the correct positioning of the ultrasound focus, high-speed imaging was first performed with a macro lens before finalising the experimental setup.

### 2.8. Loading and release of model drug Nile red using 1.1 MHz and 60 kHz ultrasound

Nile red was loaded onto hPDA NPs by mixing hPDA with Nile red dissolved in DMSO at 8 wt% ratio. The reaction mixture was allowed to incubate overnight to facilitate dye incorporation. To remove unbound Nile red, the mixture was centrifuged at 2000 rpm and the pellet was discarded. The solution was then subjected to sequential washing in a 10% ethanol : water solution at 14 000 rpm, with the supernatant discarded after each wash until it appeared clear, indicating the removal of excess dye.

To explore ultrasound-aided Nile red release, a 12–14 KDa dialysis tube was filled with 4 mL of NR@hPDA suspension (1 mg mL^−1^). The tube was then placed inside a plastic container equipped with mylar membrane (6 μm thickness; Goodfellow, UK), which served as a window for the ultrasound signal (SI, Fig. S2). This assembly was then submerged in a container filled with degassed water at 37 °C and aligned with the transducer for ultrasound exposure.

The suspension was subjected either to 1.1 MHz or 60 kHz ultrasound in a pulsed mode, with 2 min ON followed by 3 min OFF. At the 5th minute, 50 μL aliquots were collected for analysis and measured using UV-vis at 552 nm on a plate reader.

### 2.9. Ultrasound-induced drug release and viability in the biocells

The experimental setup for ultrasound-aided drug release and cell viability assessment of SN-38@hPDA was identical to that used in Nile red release study. However, instead of the container with Mylar windows, a Biocell (in-house fabricated ultrasound compatible cell culture cassette)^46^ was used to facilitate cell-based experiments (SI, Fig. S3). The Biocell was created to overcome the limitations presented by traditional cell culture plastic ware in ultrasound experiments consists It consists of a 70 mm diameter 3D printed circular frame over which mylar windows are secured to create a sterile and sealed chamber.^46^ The chamber is accessed using self-sealing septa ports within the frame that allow the addition of cells and media. The diameter of the cassette is larger than the diameter of the ultrasound field used within these experiments to prevent reflections and reverberations that could disrupt the ultrasound field incident on the cells, and the mylar is acoustically transparent such that attenuation of the field due to transmission through the window is negligible. The use of this cell culture platform allows simple and sterile maintenance of detailed characterisation of the ultrasound field so that the exact ultrasound conditions experienced by the cells can be known.

First, BxPC-3 cells were seeded onto the Mylar sheet of the Biocell with RPMI-1640 medium supplemented with 10% FBS and 0.5% pen per strep and incubated at 37 °C until reaching sufficient confluency. Once the confluency was achieved, 0.02 mg mL^−1^ hPDA was added to the medium and incubated for an additional 1 h to allow for cell acclimatisation. For loaded hPDA, concentration of SN38 was kept at 0.007 μM as it falls within the range where clear differences between the free drug and the drug-loaded particles can be observed. This concentration was selected to effectively assess the impact of ultrasound on these differences. The Biocell was then placed in the water tank and positioned at the focus of the ultrasound transducer. Cells were exposed to 1.1 MHz pulsed ultrasound for 10 min driven for 100 cycles (100 mVpp), approximate peak negative acoustic pressure of 1.2 MPa using a signal generator and power amplifier as above, after which the Biocell was returned to the incubator for a 6-hour post-treatment incubation period. Following incubation, cell viability was assessed using Alamar Blue assay.

## 3. Results and discussion

### 3.1. Synthesis and characterisation of hPDA nanocarriers

**Fig. 2:**
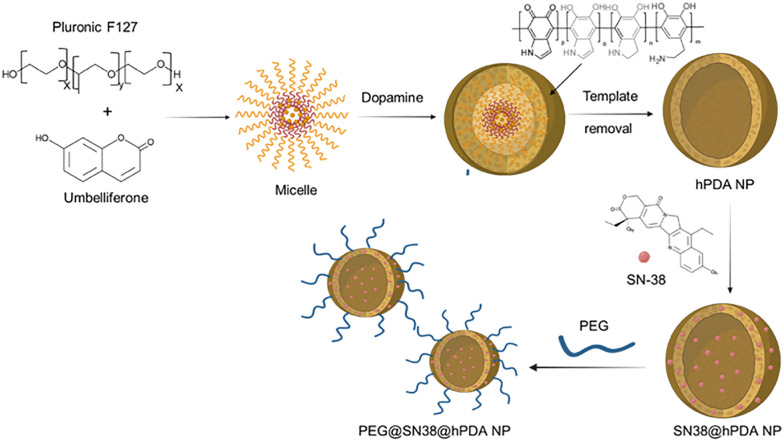
Scheme of hPDA NPs preparation, drug loading, and surface modification. Pluronic F127 and umbelliferone form micelle templates for PDA shell formation; their removal yields hollow structures, which are drug-loaded and stabilized *via* amine-PEG functionalisation.

The hollow polydopamine nanoparticles (hPDA NPs) were synthesised in aqueous solution using Pluronic F127 and umbelliferone as organic templates (Fig. 2). Umbelliferone, a hydroxycoumarin derivative, and Pluronic F127, a triblock copolymer (PEO–PPO–PEO), self-assemble into micelles due to their amphiphilic nature, serving as sacrificial templates for porous PDA formation. Pluronic F127 forms micelles above its critical micelle concentration (CMC) and critical micelle temperature (CMT), where the hydrophobic PPO cores aggregate and are stabilised by hydrophilic PEO chains in aqueous media.^47,48^ Umbelliferone, containing a hydrophobic coumarin core and a hydroxyl (–OH) group,^49^ is likely incorporated into the Pluronic F127 micelles through hydrophobic interactions with the PPO core.

The micellar architecture of Pluronic F127 provides an effective template for PDA deposition. The hydrophilic PEO corona interacts with the aqueous phase, permitting dopamine molecules to diffuse freely in solution, while the hydrophobic PPO core maintains micelle stability. Upon introduction of dopamine into an alkaline medium (typically pH 8.5–9.0), the monomer undergoes auto-oxidation to dopamine quinone, which subsequently polymerises into PDA.^43^ The resulting polymer preferentially adsorbs onto the micelle surface through hydrogen bonding, π–π stacking, and electrostatic interactions, forming a uniform PDA coating. Following polymerisation, the micellar template is removed using organic solvents and sonication, yielding hollow PDA shell.

**Fig. 3:**
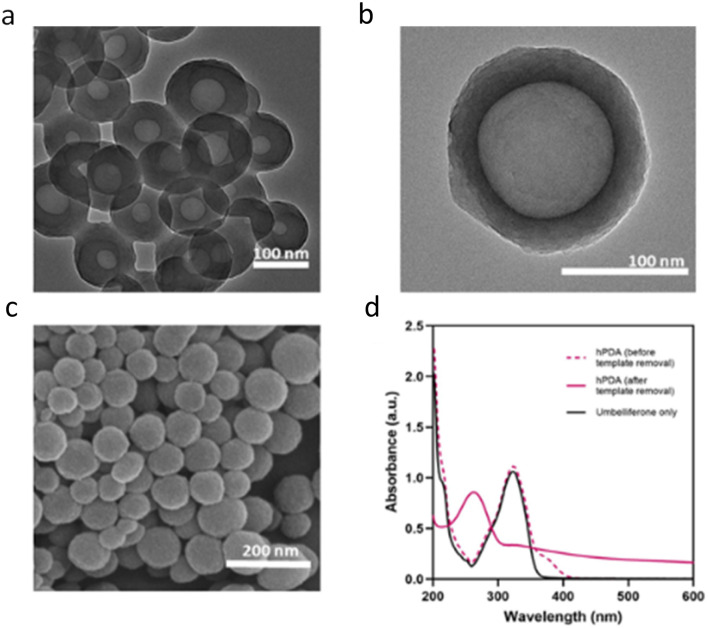
Hollow PDA (hPDA) nanoparticles. TEM images of hPDA NPs (a) and (b), SEM image of hPDA (c), and UV-vis spectra of umbelliferone template, and hPDA NPs before and after template removal (d).

Dynamic light scattering (DLS) analysis confirmed the formation of spherical hPDA particles with an average diameter of 124 ± 12 nm, slightly larger than PDA NP controls (118 ± 11 nm) synthesised *via* a previously reported dopamine polymerisation methods (SI, Fig. S4).^37^ SEM images revealed that the morphology, size, and surface roughness of hPDA NPs were comparable to those of PDA NPs.^50–52^ In contrast, TEM images clearly demonstrated the presence of a hollow core in hPDA structures (Fig. 3a and b), with some variability in wall thickness likely resulting from subtle differences in synthesis conditions and reaction timing. SEM images (Fig. 3c) further confirmed their spherical morphology and slightly rough surfaces. UV-vis spectroscopy of hPDA NPs displayed a characteristic absorption peak at ∼270 nm (Fig. 3d), while zeta potential measurements indicated a surface charge of −20.1 ± 1.3 mV, consistent with the colloidal stability and net negative charge typical for PDA NPs, attributable to the presence of surface catechol groups.

To determine the optimal synthesis time for achieving uniform and reproducible hPDA NPs, the hydrodynamic size and absorbance of the reaction mixture were monitored over time (SI, Fig. S4), and TEM was utilised to track particle morphology during the synthesis process. The data indicated that particle homogeneity, reflected by a low polydispersity index (PDI), was highest between 24 and 46 h, during which the hydrodynamic diameter ranged from 91 nm to 295 nm. Beyond 46 h, an increase in PDI suggested the initiation of particle aggregation, indicating reduced nanoparticle stability. These size measurements were obtained prior to purification and therefore include the surrounding solvation layer, accounting for the larger hydrodynamic diameters observed. TEM analysis, performed alongside DLS measurements, was subsequently used to determine particle size after washing and purification employed to determine the size of the synthesized NPs after the wash and purification steps.

The UV-vis absorbance of the NP suspension gradually increases at 450 nm over the first 46 h, consistent with the progressive formation of the of PDA layer (SI, Fig. S5).

After 54 h, a sharp rise in absorbance at 450 nm was observed, indicative of NP aggregation and correlating with the increased PDI and hydrodynamic diameter. Based on these observations, the optimal synthesis window was determined to be between 24 and 46 h, producing monodisperse and stable hPDA NPs suitable for reproducible manufacturing in subsequent studies.

UV-vis spectral analysis of the hPDA NPs after the wash steps confirmed complete removal of the umbelliferone template, as indicated by the absence of its characteristic peak at 325 nm (Fig. 3d). Template removal is achieved through sonication with ethanol–acetone mixture, which disrupts micelle stability by altering the solvation dynamics of both Pluronic F127 and umbelliferone.^53,54^ Acetone, a polar aprotic solvent, has a strong affinity for PPO region of F127, thereby reducing the hydrophobic effect that drives micelle formation and destabilising the core.

Ethanol, a polar protic solvent, can solvate both PEO and PPO blocks, further weakening the self-assembled micellar architecture. This solvent combination effectively reduces the solubility contrast between the hydrophilic and hydrophobic domains of Pluronic F127, resulting in complete micelle disassembly.

Following template removal, a new absorption peak emerged at 263 nm. This peak, previously obscured by the strong absorbance of umbelliferone, is likely attributable to catechol groups in hPDA, a spectral feature also reported for PDA and dopamine.^55,56^ Notably, different hPDA concentrations were used for these measurements to account for the high absorbance of umbelliferone prior to removal. To avoid signal saturation, pre-purification UV-vis measurements (before template removal) were performed at concentrations 10× lower than those used for post-purification measurements. Following template removal and multiple washing steps, the resulting hPDA NP suspensions, free of unbound umbelliferone, enabled accurate spectral characterisation of the purified NPs.

### 3.2. hPDA cell uptake and drug loading

For cell studies, hPDA NPs were functionalised with methoxy-PEG-amine *via* a Schiff base reaction or Michael addition, resulting in PEGylated surfaces. PEGylation is a crucial modification that enhances the colloidal stability of hPDA NPs by reducing aggregation in biological media. In addition, the PEG coating improves biocompatibility, reduces non-specific protein adsorption, and limits immune recognition, thereby prolonging NP circulation time.^57^ Although PEG is neutral, the zeta potential of PEGlyated hPDA NPs remained moderately negative (20 mV), which can be attributed to residual surface catechol groups not fully shielded by PEG chains. PEG primarily enhances colloidal stability through steric hindrance rather than electrostatic effects, and even NPs with slightly negative surface potentials can exhibit excellent stability and stealth behaviour under physiological conditions.

Following PEGylation, we evaluated the cytotoxicity of both hPDA and PDA nanocarriers. Previous studies, including our own, have demonstrated that PDA-based nanostructures, which mimic the natural pigment melanin, exhibit high biocompatibility and are well-suited for drug delivery applications.^37^ Consistent with these findings, cell viability assays in BxPC-3, PANC-1, and a healthy pancreatic cell line human pancreatic stallate cell (hPSC) confirmed the negligible cytotoxicity of hPDA NPs, further supporting their suitability as a safe and effective drug delivery platform.

The internalisation of NPs into diseased cells is a key indicator of their potential efficacy in drug delivery. This process is governed by the interplay between the physicochemical properties of the NPs and the biological characteristics of the target cell. Typically, cellular uptake occurs *via* endocytosis, with the NPs subsequently localising within lysosomes.^58–60^

To investigate hPDA NP uptake, we performed confocal imaging using rhodamine-labelled hPDA and PDA NPs (Rh@hPDA and Rh@PDA) in BxPC-3 and PANC-1 human pancreatic ductal adenocarcinoma (PDAC) cell lines. These epithelial-like cells adhere readily to culture vessels, making them suitable for *in vitro* studies. Genetically, they exhibit distinct mutational profiles representative of key molecular alterations in PDAC. BxPC-3 cells harbour a mutation in in the p53 tumour suppressor gene^61^ but, notably, lack the common *KRAS* mutation, making them a representative model for non-*KRAS*-driven tumorigenesis. In contrast, PANC-1 cells carry mutations in both *KRAS* and *p53.* These genetic differences make BxPC-3 and PANC-1 valuable complementary models for examining *KRAS*-independent *vs. KRAS*-driven tumour progression and for assessing differential therapeutic responses.

**Fig. 4:**
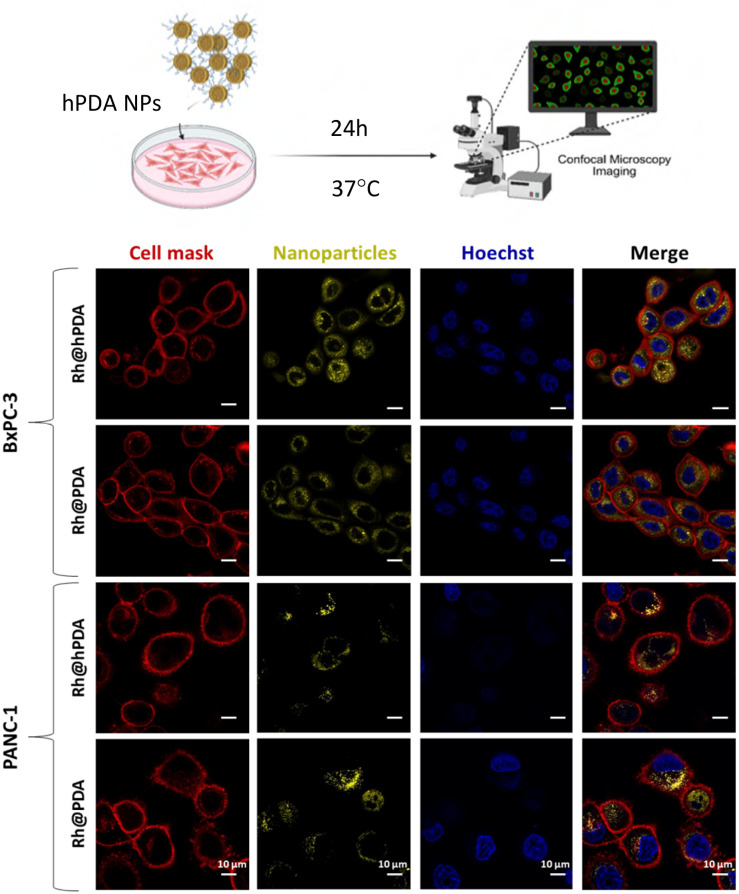
Schematic of PDAC cells incubated with rhodamine-labelled NPs for 24 h at 37 °C and confocal images showing uptake of rhodamine-labelled hPDA (Rh@hPDA) and PDA (Rh@PDA) in BxPC-3 and PANC-1 cells. Cell membrane is shown in red, nuclei blue, and NPs yellow.

As shown in Fig. 4, both Rh@hPDA and Rh@PDA NPs were efficiently internalised into BxPC-3 and PANC-1 cells within 24 h. This demonstrates the capacity of hPDA NPs to cross the cellular membrane and reach the intracellular environment, a critical requirement for effective drug delivery. The uptake behaviour and intracellular localisation of Rh@hPDA NPs were comparable to those of conventional PDA NPs, suggesting that both utilise similar endocytic pathways.

Following the uptake studies, hPDA NPs were employed as nanocarriers for SN-38, the active metabolite of irinotecan, a chemotherapeutic agents used to treat cancer including lung, ovarian, gastric, and cervical malignancies. SN-38 inhibits topoisomerase I, resulting in DNA damage and subsequent cell death.^62,63^ Although over 1000 times more potent than irinotecan, SN-38 has not been approved for clinical use due to its poor water solubility, high systemic toxicity, low stability, and rapid metabolism and clearance.^64–66^

These limitations make SN-38 an excellent candidate for nanoparticle-based delivery systems, which can improve solubility, enhance tumour targeting, and reduce off-target toxicity.

To evaluate the therapeutic activity of hPDA-encapsulated SN-38 relative to the free drug, MTS cell viability assays were performed on two pancreatic cell lines, using drug-free hPDA and PDA NPs as controls. SN-38 was successfully loaded onto hPDA NPs with a loading capacity of 8.1 ± 1.4% (SI, Table S2). DLS and zeta potential measurements confirmed that drug loading did not markedly alter the particle size (151 ± 11 nm *vs.* 124 ± 12 nm for unloaded hPDA) or surface charge (−21.8 ± 2.2 mV *vs.* −20.1 ± 1.3 mV), indicating good formulation stability.

**Fig. 5:**
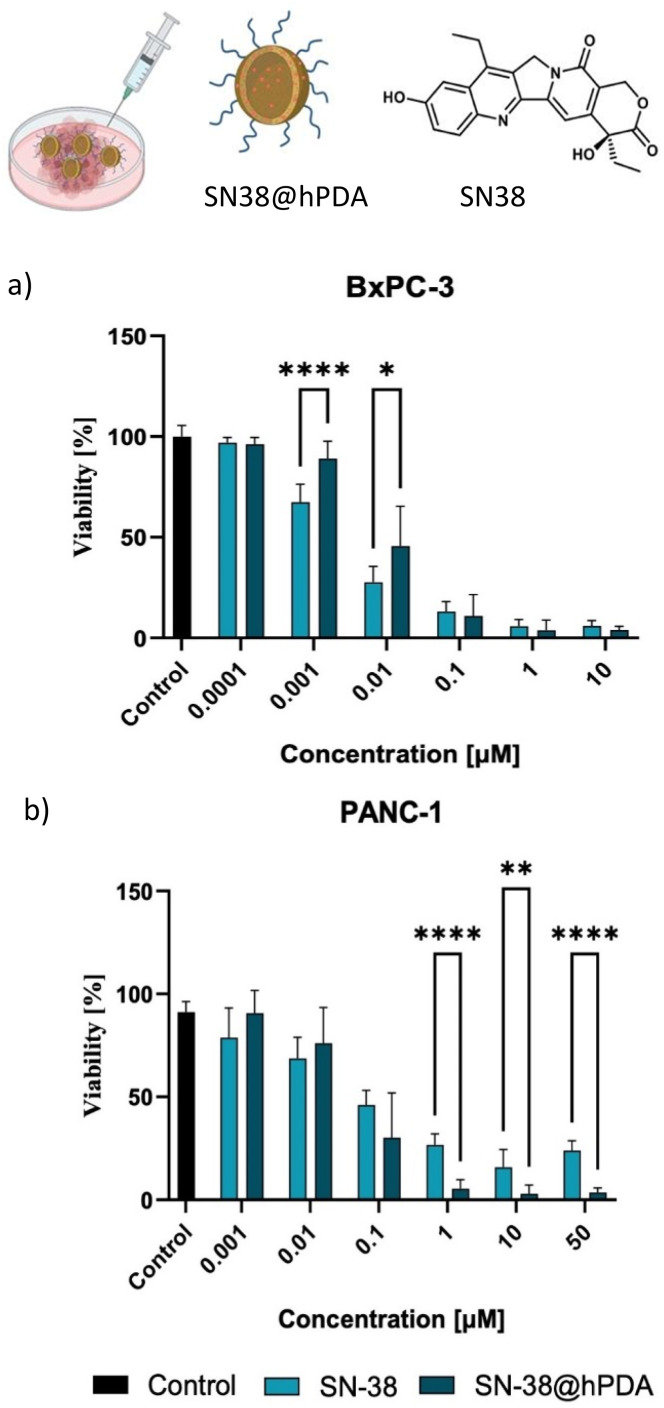
Cytotoxicity of SN-38 loaded nanocarriers. Drug-loaded NPs are added to BXPC-3 (a) and PANC-1 (b) cells and cell viability monitored at different concentrations of free drug or SN-38@hPDA NPs. Asterisks indicate statistical significance where: *P < 0.05, **P < 0.01, ***P < 0.001, ****P < 0.0001.

As shown in Fig. 5 SN-38 loaded hPDA NPs exhibited dose-dependent cytotoxicity after 72 h, comparable to or exceeding that of free SN-38, particularly at higher concentrations. In BxPC-3 cells, SN-38@hPDA demonstrated significantly greater cytotoxicity than free SN-38 at 0.1 μM and 10.0 μM, suggesting improved drug delivery and intracellular retention. The effect was even more pronounced in PANC-1 cells, where SN-38@hPDA consistently outperformed free SN-38 across a wide concentration range (0.1 μM to 50.0 μM), with highly significant differences observed (SI, Table S1). These findings indicate that the hPDA nanocarrier system not only efficiently delivers SN-38 to pancreatic cancer cells but also enhances its cytotoxic effects, particularly in PANC-1 cells, which harbours a *KRAS* mutation associated with therapeutic resistance. Cell viability in BxPC-3 and PANC-1 cells was also evaluated following exposure to free PDA, hPDA, and SN-38@PDA for comparative analysis (SI, Fig. S6 and S7).

Overall, the results confirm hPDA NPs as a promising drug delivery platform for PDAC therapy, warranting further mechanistic studies and *in vivo* evaluation to fully establish their therapeutic potential. Building on these encouraging results, we next explored the feasibility of employing hPDA for on-demand, ultrasound-guided drug release.

### 3.3. Ultrasound-induced cavitation of hPDA NPs

Ultrasound-responsive materials can undergo acoustic cavitation, a phenomenon in which gas bubbles rapidly oscillate and collapse under the influence of ultrasound waves. This can mechanically disrupt tissues, increase cell membrane permeability, and promote intracellular drug uptake.^25^ It can also trigger payload release by breaking carrier structures and enhance tissue penetration *via* microstreaming and acoustic radiation forces.^21,22^

Together, these effects enable localised delivery and can significantly improve therapeutic outcomes. Consequently, microbubbles (MBs), clinically approved ultrasound contrast agents,^67,68^ have also been adapted as drug delivery vectors,^21^ such as lipid-based MBs carrying doxorubicin for pancreatic carcinoma treatment.^69^ However, limitations including large size, composition, short circulation time, and structural instability have driven the development of nanoparticles for ultrasound-mediated delivery, offering improved targeting and tissue penetration, as well as the more favourable biodistribution.^70,71^

Taking these advantages into account, we investigated the ultrasound responsiveness of hPDA NPs and evaluated their potential to enhance cytotoxicity under ultrasound exposure. Specifically, we examined whether ultrasound stimulation could amplify therapeutic efficacy, thereby improving targeted drug delivery and cancer treatment outcomes.

The ultrasound responsiveness of hPDA was first assessed using a custom experimental setup equipped with a high-speed camera to visualize cavitation events, indicative of gas escape and shell disruption. Various driving pressures and frequencies were tested during the initial phase. We determined that a 1.1 MHz ultrasound signal with a driving voltage of 80 mVpp, corresponding to an approximate peak negative acoustic pressure of 1.2 MPa, represented the minimum threshold required to induce cavitation in hPDA.

Stable cavitation, a state in which most bubbles undergo repeated radial expansion and contraction with minimal destruction during short excitation cycles, typically occurs at pressures around 0.25 MPa.^21,72^ In contrast, inertial cavitation involves rapid expansion followed by violent collapse, releasing the gas core.^73^ After collapse, residual gas may continue to oscillate or fragment, forming a cloud of acoustically active microcavities.^73,74^

Commercial MBs (∼3 μm in diameter) exhibit stable cavitation at 0.25 MPa, a mix of stable and inertial cavitation at 0.5 MPa, and predominantly inertial cavitation at 1 MPa.^21,71^ However, hPDA NPs, with a markedly smaller size (∼124 nm) compared to MBs such as SonoVue (∼2.5 μm, used for contrast-enhanced ultrasound in the UK),^74^ is expected to require a higher acoustic pressure to induce cavitation. In MBs, ultrasound effects are amplified at their resonance frequency, which depends on bubble size, surrounding fluid properties, and shell composition.^75,76^ Because hPDA NPs are much smaller, they exhibit higher resonance frequencies and are not easily excited under standard ultrasound conditions, necessitating greater acoustic pressures for effective disruptions.^77^

**Fig. 6:**
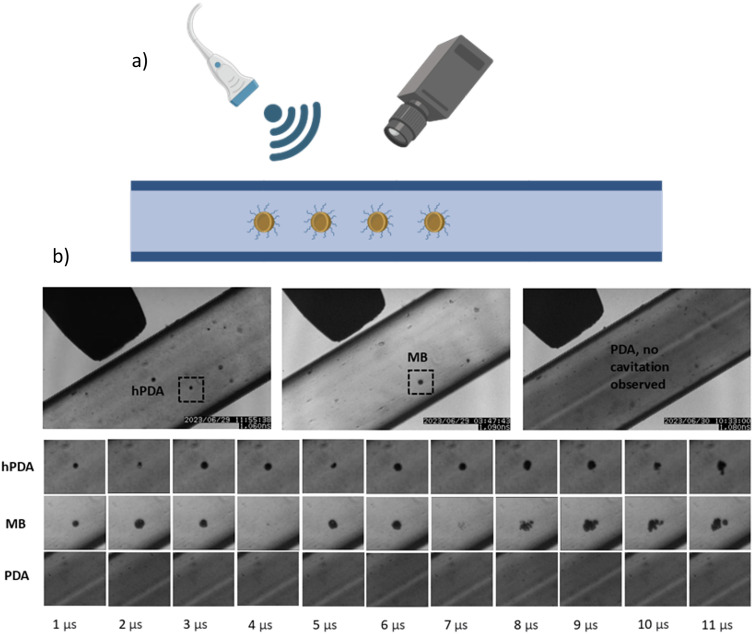
Cavitation of different types of particles under ultrasound application. (a) Illustration of the experimental set-up; NPs are injected into a 500 μm polycarbonate capillary and exposed to 100 cycles at 1.1 MHz ultrasound. Cavitation events are captured using a high-speed camera. (b) Cavitation clouds captured over 11 μs for hollow polydopamine nanoparticles (hPDA) compared to commercially available microbubbles (MB) and polydopamine (PDA) controls.

A comparison of hollow hPDA NPs, commercial MBs, and PDA NP controls is shown in Fig. 6, hPDA NPs underwent cavitation upon ultrasound exposure, whereas control PDA NPs showed no such activity. The cavitation cloud produced by hPDA is comparable in appearance to that of MBs.

However, it is important to note that the cloud size does not directly reflect the size of the cavitating particles. As ultrasound intensity increases, oscillation amplitude grows until the inward-moving fluid wall gains enough inertia to prevent reversal when the acoustic pressure phase changes.^16,21,25^ The wall then continues to compress the gas within the bubble to a very small volume, generating high pressures and temperatures, a phenomenon known as transient (inertial) or collapse cavitation. This process can damage cells and vesicles through intense shear stresses, shockwaves produced during collapse, and free radical generation.^21,72^ Collapse events also produce small bubbles that act as cavitation nuclei, which can grow and collapse repeatedly.^19,21,78^ Consequently, the initial particle size is not a direct determinant of cavitation cloud size. The ability of hPDA NPs to cavitate at 1.1 MHz prompted further investigation into ultrasound-triggered drug release and cytotoxic effects.

### 3.4. Ultrasound-triggered release of model drug

To investigate ultrasound-guided drug release, Nile red, a hydrophobic compound widely used as a model drug, was selected due to tits ease of spectroscopic detection. After loading it into hPDA NPs, release was monitored over 55 min under an ultrasound regimen of 2 min ON, 3 min OFF, with samples collected every 5 min. The OFF period allowed diffusion across the cellulose membrane enclosing the NP solution, enabling mixing of free Nile red into the surrounding water–ethanol medium (SI, Fig. S2). Drug release was tested using both clinically relevant 1.1 MHz ultrasound and a higher-energy 60 kHz frequency, the latter serving as a positive control owing to its strong mechanical effects on NPs.

**Fig. 7:**
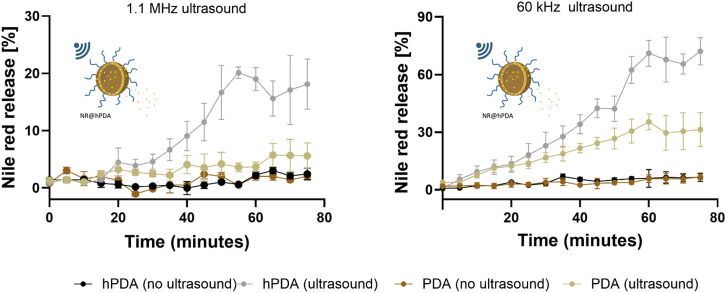
Drug release profile of model hydrophobic drug Nile red from hPDA over 75 minutes with 1.1 MHz and 60 kHz ultrasound exposure.

As shown in Fig. 7, ultrasound exposure markedly enhanced Nile red release from hPDA NPs, with some release also detected from PDA at lower frequencies. Notably, 60 kHz ultrasound resulted in a 54% increase in Nile red release from hPDA NPs over 55 min, compared to only 19% increase for PDA NPs under the same conditions.

This difference is likely due to the enhanced cavitation activity of hPDA, arising from the inherent presence of cavitation nuclei in its hollow structure, which promotes cavitation behaviour that enhances drug release. At the given pressure, lower ultrasound frequencies have a higher mechanical index, making them more efficient at inducing mechanical effects.^79^ This explains the stronger impact of 60 kHz ultrasound, frequencies at this range are typically used in ablative and cleaning applications, with laboratory ultrasonic baths commonly operating at 40–60 kHz.^80^ Moreover, lower frequencies experience less attenuation, allowing deeper penetration into tissue. Thus, 60 kHz exposure is expected to induce highly disruptive sonication, producing ultrasound fields that prolong exposure of particles to the peak negative pressure phase of the waves, resulting in high energy deposition and mechanical disruption. This effect facilitates Nile red dissociation from PDA NPs.

In contrast, 1.1 MHz ultrasound, within the range commonly used for medical imaging and certain therapeutic applications such as joint pain relief and osteoarthritis,^77^ also proved effective in triggering Nile red release but from hPDA NPs only. These findings confirm our hypothesis that ultrasound is a promising trigger for the controlled release of therapeutic agents from hPDA NPs. Building on this, we next investigated ultrasound-mediated release of SN-38 and evaluated its cytotoxicity in a pancreatic cancer cell line.

### 3.5. Ultrasound-triggered release and cytotoxicity of SN-38@hPDA NPs

**Fig. 8:**
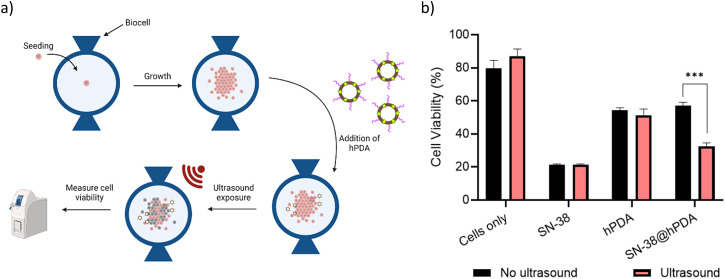
Cytotoxicity of SN38@hPDA NPs under ultrasound exposure. (a) Scheme of the Biocell experimental set up. (b) Cell viability of BxPC-3 cells treated with free drug, unloaded hPDA, and SN-38@hPDA with and without 1.1 MHz ultrasound exposure. Asterisks indicate statistical significance where: *P < 0.05, **P < 0.01, ***P < 0.001, ****P < 0.0001. SN-38 concentration used was kept at 0.007 μM.

To explore the cytotoxicity of drug-loaded hPDA NPs in absence and presence of ultrasound, the BxPC-3 cell line was selected for its high sensitivity to SN-38, enabling detection of small changes in cell viability. Cells were cultured to the desired confluency within an in-house designed device, the Biocell (SI, Fig. S8 and Fig. 8). SN-38@hPDA NPs were then introduced and incubated for 1 h to allow acclimatisation before 1.1 MHz ultrasound exposure. Cell viability was assessed using the Alamar Blue assay, in which reduced signal intensity indicated decreased cell viability. Alamar Blue contains resazurin, a blue, non-fluorescent compound that is reduced by metabolically active cells to resorufin, a pink and fluorescent product. The extent of this colorimetric and fluorometric change directly correlates with viable cell number, as non-viable cells lack the metabolic activity to convert resazurin. Because the assay is non-toxic, it permits continuous monitoring of cell viability over time without compromising cell integrity.^81^

As shown in Fig. 8, free SN-38 shows significant toxicity regardless of ultrasound. In contrast, SN-38@hPDA resulted in a significant reduction in cell viability under ultrasound exposure compared to the unexposed group, confirming the 1.1 MHz ultrasound enhances the cytotoxicity of SN-38@hPDA NPs. In contrast, free SN-38 shows significant toxicity regardless of ultrasound. This effect may arise from (i) ultrasound-induced drug release, as demonstrated using the Nile red model and/or (ii) ultrasound-facilitated uptake of hPDA NPs and free drug by cells. Both stable and inertial cavitation can transiently increase cell membrane permeability, a process known as sonoporation, thereby enhancing drug delivery and chemotherapeutic efficacy.

It is anticipated that the observed effects arise from a combination of nanoparticle endocytosis and intracellular uptake, along with extracellular drug release and its associated cytotoxicity. Determining the relative contribution of these mechanisms will be an important focus of future studies. Previous studies have shown that combining chemotherapy with ultrasound-mediated delivery can markedly improve tumour regression and survival in animal models of pancreatic cancer.^16,25,78,82^ Building on these findings, our future studies will investigate the effects of ultrasound in 3D pancreatic cancer models, and in *in vivo* mice models.

## 4. Conclusions

In this study, we developed and evaluated hollow polydopamine (hPDA) nanoparticles (NPs) as a platform for ultrasound-enhanced drug delivery. The synthesised hPDA NPs (124 ± 12 nm) were confirmed to have a hollow core, making them well suited for therapeutic payload encapsulation. hPDA efficiently loaded the chemotherapeutic agent SN-38 and exhibited cytotoxicity comparable to free SN-38 against Pancreatic Ductal Adenocarcinoma (PDAC) cell lines, BxPC-3 and PANC-1. Cellular uptake studies confirmed efficient internalisation of hPDA by cancer cells, a prerequisite for effective NP-based delivery.

Ultrasound-triggered release experiments demonstrated the strong acoustic responsiveness of hPDA. Compared with solid PDA NPs, hPDA exhibited a 54% increase in drug release under 60 kHz ultrasound and a 19% increase at 1.1 MHz, alongside a 20% greater reduction in cell viability under ultrasound exposure, demonstrating strong synergy between acoustic cavitation and polymer shell rupture.

Collectively, this work establishes a melanin-inspired, ultrasound-responsive hollow polydopamine (hPDA) nanoparticle platform that unites biocompatible synthesis, nanoscale precision, and externally triggered therapy within a single system. Synthesised using non-toxic template (umbillferone), these nanoparticles exhibit excellent acoustic sensitivity, high drug-loading capacity, and robust biological compatibility, enabling spatiotermporally controlled and non-invasive drug release. The cavitation-induced echogenic response further introduces a unique capability for real-time, *in situ* monitoring of therapeutic delivery.

By overcoming key limitations of traditional microbubble and solid PDA systems, this hPDA platform offers a clinically relevant, multifunctional approach for next-generation cancer therapy-particularly for treating dense and treatment-resistant tumours such as PDAC. As a non-invasive modality, therapeutic ultrasound is a suitable alternative to intratumoral administration of drugs, considered as an option for hard-to-treat tumours. Therefore, presented results provide a strong foundation for the continued development and future translation of ultrasound-responsive nanotherapeutics.

## Conflicts of interest

There are no conflicts to declare.

## Supplementary Material

NH-011-D5NH00297D-s001

## Data Availability

The data supporting this article (details of the ultrasound setup, Biocell design, and additional data concerning drug loading and nanoparticle properties) have been included as a part of the supplementary information (SI). See DOI: https://doi.org/10.1039/d5nh00297d.
