# Supplementary material for: Hollow-core polydopamine nanocarriers for ultrasound-enhanced drug delivery
Source: Nanoscale Horiz. 2025 Nov 6;11(1):211–24. doi: 10.1039/d5nh00297d (PMC12599296; doi:10.1039/d5nh00297d)
Supplement: NH-011-D5NH00297D-s001 [file NH-011-D5NH00297D-s001.pdf]

Supplementary Information (SI) for Nanoscale Horizons.
This journal is © The Royal Society of Chemistry 2025

Supplementary Information

Hollow-Core Polydopamine Nanocarriers for Ultrasound-Enhanced Drug Delivery

Swetha Lingamgunta[1a], Chitra Yadav[1a], Andrea Orthodoxou[2b], Lauren Gilmour[3b], Matthew Ellis[4c], Hildegard Metzger[2c], Andrea Bistrovic Popov[1c], Helen Mulvana[2d], Ljiljana Fruk[1d&]

[1] Department of Chemical Engineering and Biotechnology, University of Cambridge, Cambridge, UK

[2] Department of Biomedical Engineering, University of Strathclyde, Glasgow, UK

[3] James Watt School of Engineering, University of Glasgow, Glasgow, UK

[4] Department of Physics, University of Cambridge, Cambridge, UK

[&]Senior author

[a] These authors contributed equally to this work.

[b] These authors contributed equally to this work.

[c] These authors contributed equally to this work.

[d] These authors contributed equally to this work.

[&] Corresponding author

Email: lf389@cam.ac.uk

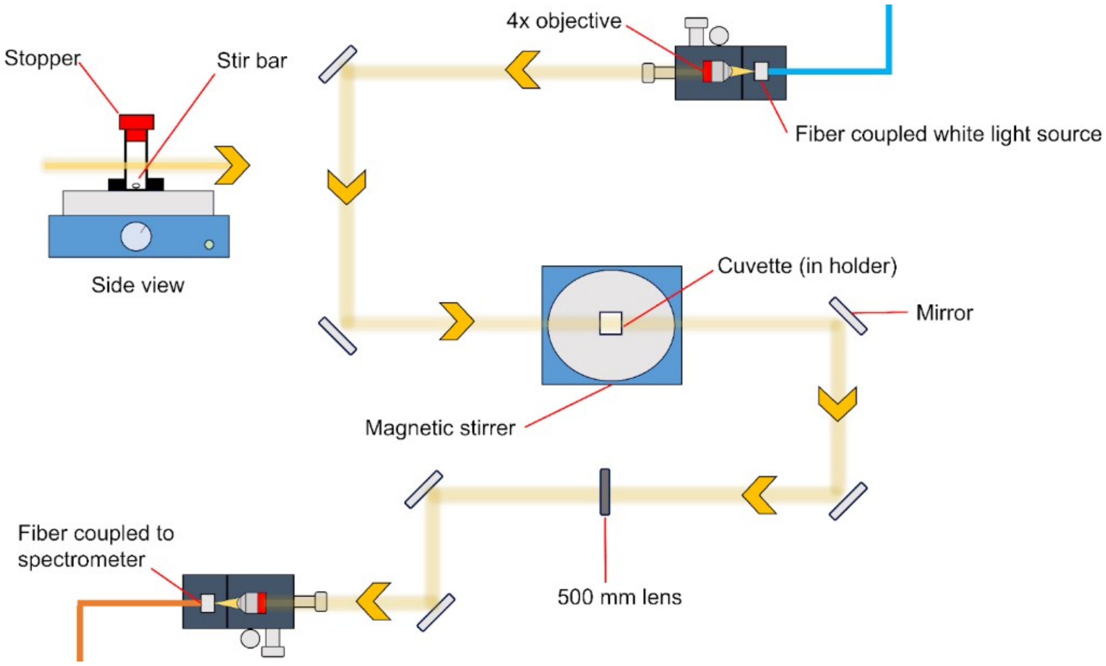

SI Figure S1 Custom set-up for the UV-VIs measurements of hPDA synthesis over 72 h. The set up shows a DH-2000 Deuterium-Tungsten Halogen UV-Vis-NIR Light Source (Ocean Insight), with light coupled into an Ocean Optics QE65000 spectrometer.

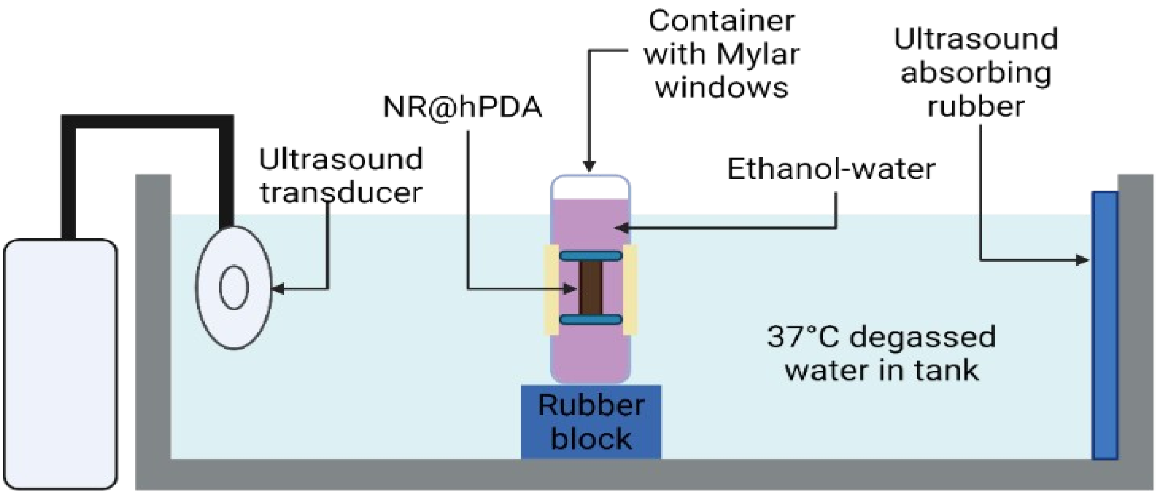

SI Figure S2 Experimental set up to measure model drug, Nile red, release from hPDA under exposure to clinically relevant 1.1 MHz ultrasound.

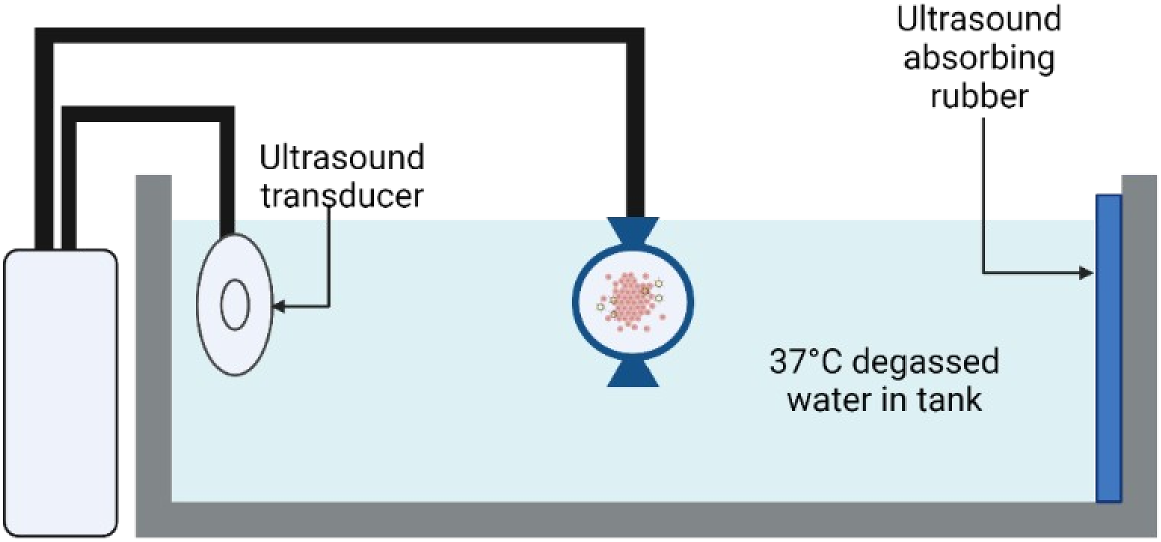

SI Figure S3 Experimental set-up to measure cytotoxicity of SN-38@hPDA to BxPC-3 under exposure to clinically relevant 1.1 MHz ultrasound.

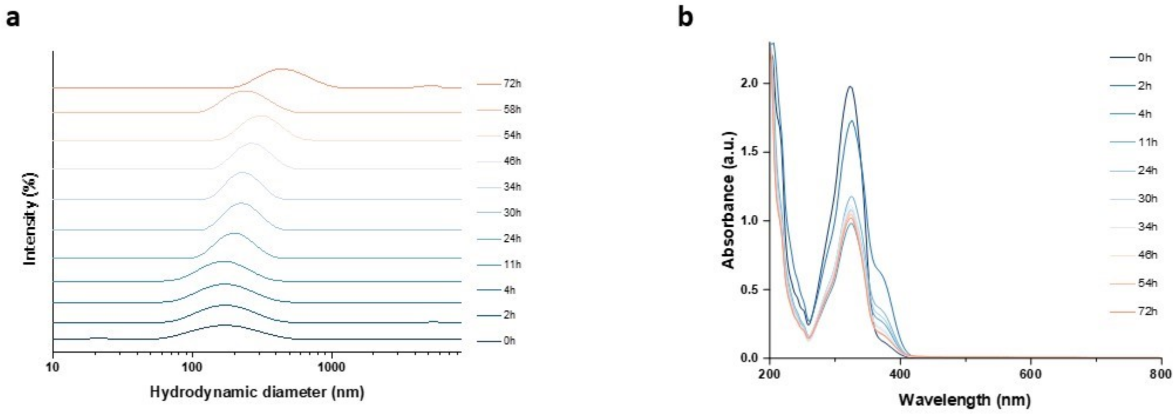

SI Figure S4 a) DLS data over 72 h showing hydrodynamic size of the hPDA NPs in the reaction mixture. b) UV-Vis data of the reaction mixture over 72 h.

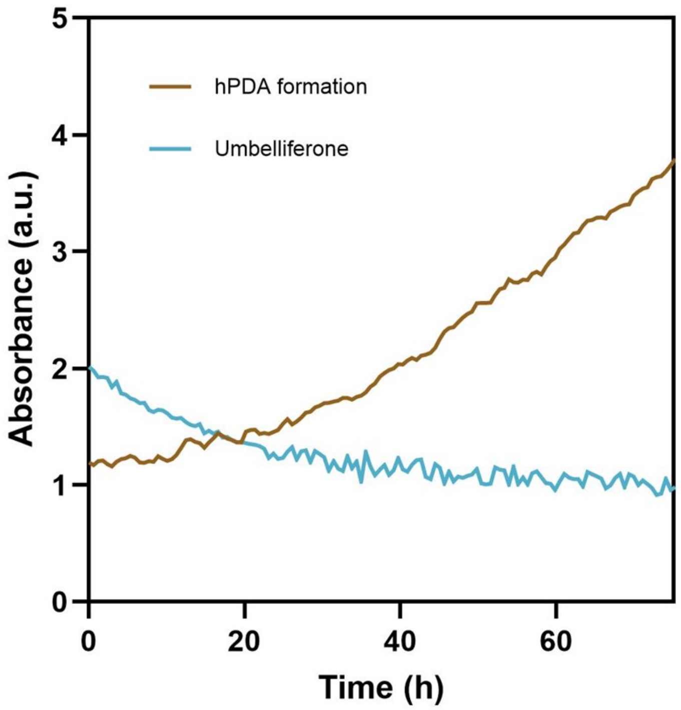

SI Figure S5 UV-Vis spectra over 72 h at 325 nm (umbelliferone) and 450 nm (showing formation of PDA coating on micelle).

SI Table 1 Table showing calculated IC50 values for SN-38 and SN-38@PDA for both BxPC-3 and PANC-1.

Cell line	IC_{50} values (nM)		
	SN-38	SN-38@PDA	SN-38@hPDA
BxPC-3	68.3	79.5	10.8
PANC-1	115.9	148.1	84.6

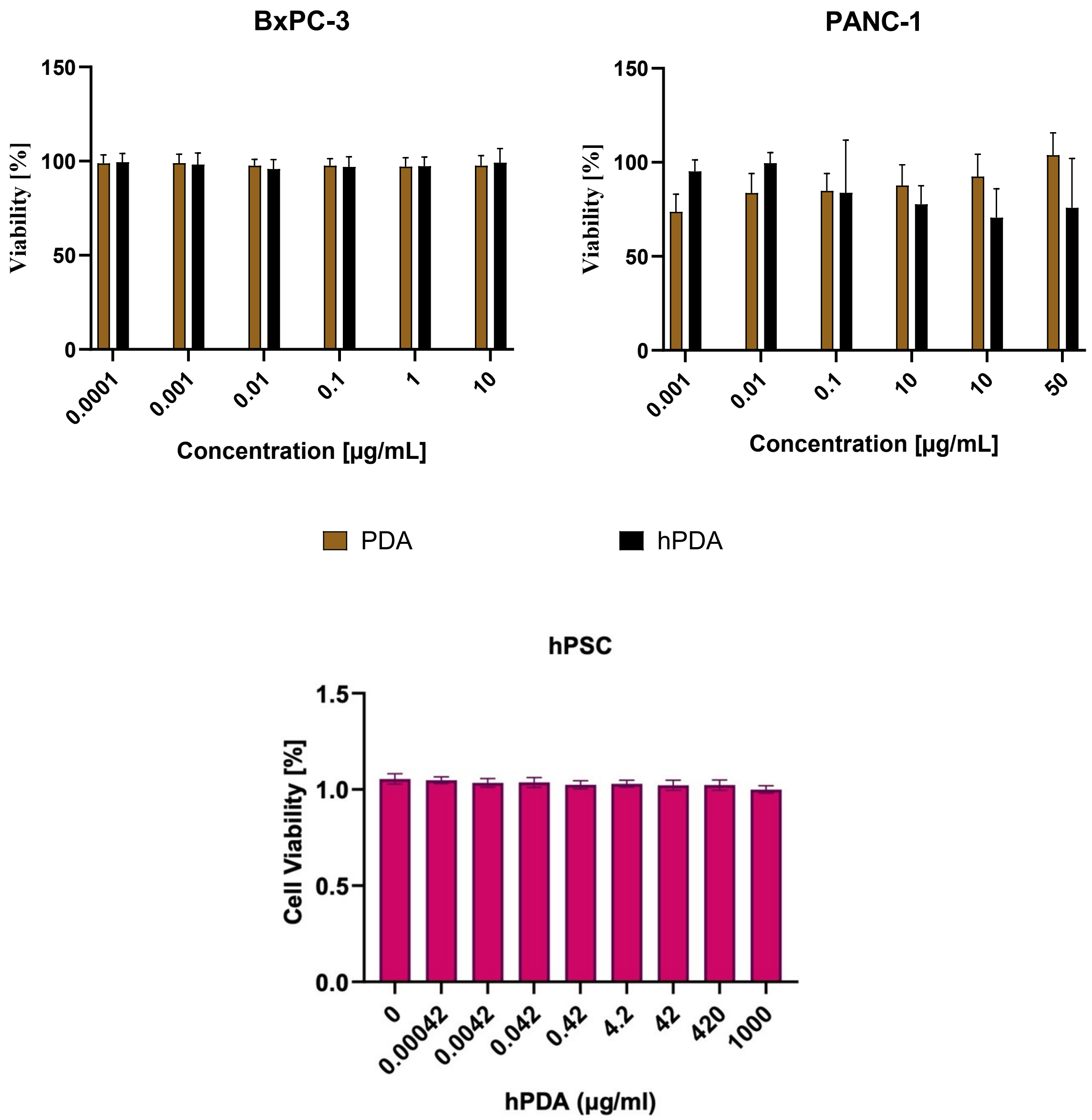

SI Figure S6 Cell viability of pancreatic cancer cells BxPC-3 and PANC-1 cells when exposed to varying concentrations of PDA and hPDA NPs for 72 hours. In addition, healthy pancreatic stellate cell hPSC was used to explore hPDA NP toxicity indicating no toxic effects of unloaded NPs. Data are presented as mean ± standard deviation, showing that neither NP formulation exhibited significant cytotoxicity across the tested concentrations.

SI Table S2: Loading capacity and loading efficiency of SN38 drug and the raw data used for calculation

	SN-38 loading capacity %	SN-38 Loading efficiency %
**PDA**	7.2 ± 1.0	28.8 ± 4.2
**hPDA**	8.2 ± 1.4	32.8 ± 5.5

	Raw absorbance values	Calculated concentration (mg/mL)	Adjusting for the 10uL in 1mL dilution for taking the measurement (mg/mL)	Concentration of sample (mg/mL) with respect to the NP	SN-38 mg/mL	Loading capacity	Loading efficiency
PDA	0.4101	0.004617413	0.461741259	6	0.076956876	7.6956876	30.7827506
	0.3416	0.003602515	0.360251523	6	0.06004192	6.004192	24.0167682
	0.4188	0.004746312	0.474631196	6	0.079105199	7.9105199	31.6420797
					Mean	**7.2034665**	**28.8138662**
					SD	**1.0441421**	**4.17656838**
hPDA	0.3717	0.004048477	0.404847742	6	0.067474624	6.7474624	26.9898495
	0.4391	0.005047077	0.504707716	6	0.084117953	8.4117953	33.6471811
	0.4819	0.005681203	0.568120281	6	0.094686714	9.4686714	37.8746854
					Mean	**8.2093097**	**32.8372387**
					SD	**1.3718582**	**5.48743276**

	Stock (NP conc, mg/mL) C1	Stock (drug conc, ug/mL) C1	V1	C2 = final concentration we want uL	V2 = final volume of the solution uL
SN38		10	1.4	0.0028	5000
SN38+NP	2	0.28	50	0.0028	5000
NP	2		50	0.02	5000

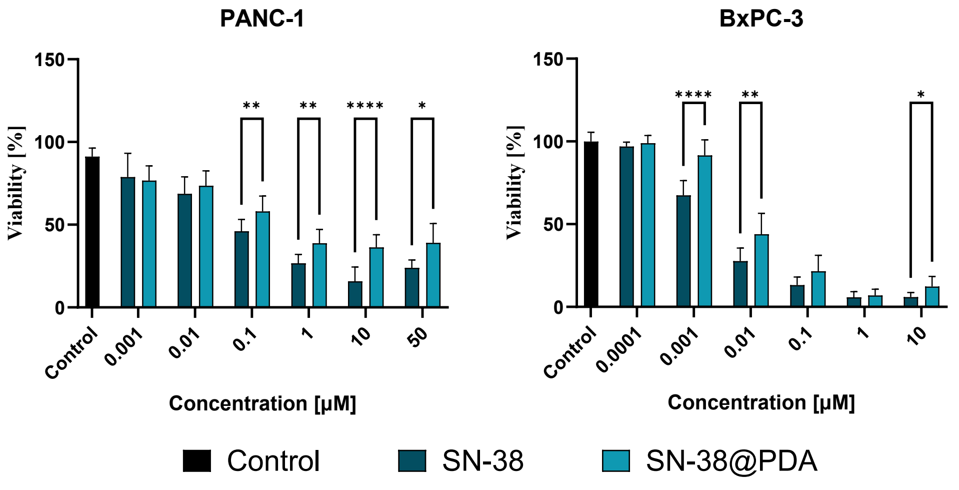

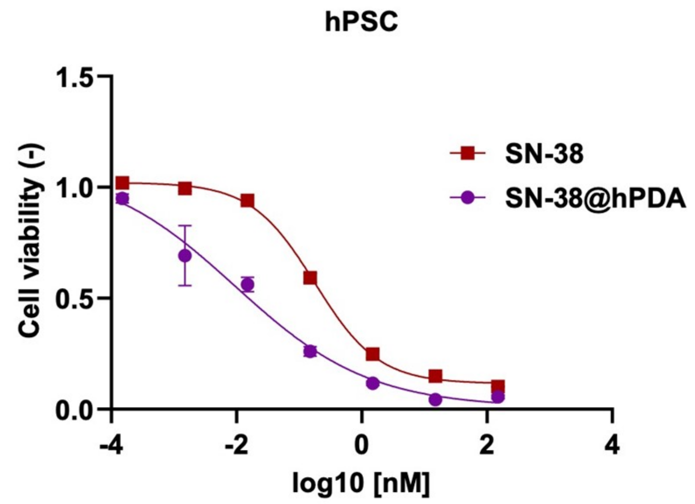

SI Figure S7 Cell viability of BxPC-3 , PANC-1 and hPSC cells when exposed to SN-38 and SN-38@hPDA. Asterisks indicate statistical significance where: *P < 0.05, **P < 0.01, ***P < 0.001, ****P < 0.0001.

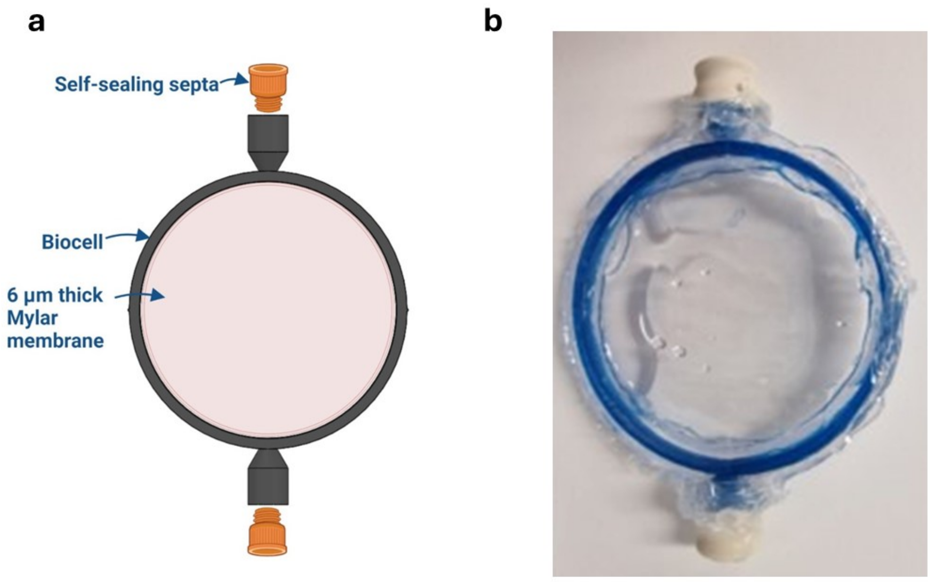

SI Figure S8 a) Diagram of the Biocell. b) Photograph of the Biocell.
